# Development and application of a machine learning-based predictive model for obstructive sleep apnea screening

**DOI:** 10.3389/fdata.2024.1353469

**Published:** 2024-05-16

**Authors:** Kang Liu, Shi Geng, Ping Shen, Lei Zhao, Peng Zhou, Wen Liu

**Affiliations:** ^1^Department of Otolaryngology, Head and Neck Surgery, Affiliated Hospital of Xuzhou Medical University, Xuzhou, China; ^2^Artificial Intelligence Unit, Department of Medical Equipment Management, Affiliated Hospital of Xuzhou Medical University, Xuzhou, China

**Keywords:** machine learning, obstructive sleep apnea, prediction model, LightGBM, Random Forest

## Abstract

**Objective:**

To develop a robust machine learning prediction model for the automatic screening and diagnosis of obstructive sleep apnea (OSA) using five advanced algorithms, namely Extreme Gradient Boosting (XGBoost), Logistic Regression (LR), Support Vector Machine (SVM), Light Gradient Boosting Machine (LightGBM), and Random Forest (RF) to provide substantial support for early clinical diagnosis and intervention.

**Methods:**

We conducted a retrospective analysis of clinical data from 439 patients who underwent polysomnography at the Affiliated Hospital of Xuzhou Medical University between October 2019 and October 2022. Predictor variables such as demographic information [age, sex, height, weight, body mass index (BMI)], medical history, and Epworth Sleepiness Scale (ESS) were used. Univariate analysis was used to identify variables with significant differences, and the dataset was then divided into training and validation sets in a 4:1 ratio. The training set was established to predict OSA severity grading. The validation set was used to assess model performance using the area under the curve (AUC). Additionally, a separate analysis was conducted, categorizing the normal population as one group and patients with moderate-to-severe OSA as another. The same univariate analysis was applied, and the dataset was divided into training and validation sets in a 4:1 ratio. The training set was used to build a prediction model for screening moderate-to-severe OSA, while the validation set was used to verify the model's performance.

**Results:**

Among the four groups, the LightGBM model outperformed others, with the top five feature importance rankings of ESS total score, BMI, sex, hypertension, and gastroesophageal reflux (GERD), where Age, ESS total score and BMI played the most significant roles. In the dichotomous model, RF is the best performer of the five models respectively. The top five ranked feature importance of the best-performing RF models were ESS total score, BMI, GERD, age and Dry mouth, with ESS total score and BMI being particularly pivotal.

**Conclusion:**

Machine learning-based prediction models for OSA disease grading and screening prove instrumental in the early identification of patients with moderate-to-severe OSA, revealing pertinent risk factors and facilitating timely interventions to counter pathological changes induced by OSA. Notably, ESS total score and BMI emerge as the most critical features for predicting OSA, emphasizing their significance in clinical assessments. The dataset will be publicly available on my Github.

## 1 Introduction

Obstructive sleep apnea (OSA) is characterized by recurrent apnea and hypoventilation during sleep, leading to multi-organ and multi-system damage. This condition is associated with various health issues such as hypertension, coronary artery disease, arrhythmia, heart failure, stroke, cognitive dysfunction, and type 2 diabetes, bringing a significant economic burden to patients and society (Javaheri et al., 2017; Leng et al., 2017; Tan et al., 2018). As living standards improve and lifestyles change, the prevalence of OSA is on the rise globally. A study on the global prevalence of OSA revealed that approximately 1 billion people suffer from OSA, with some countries experiencing prevalence rates exceeding 50%. Notably, China, with its large population base, carries the highest number of patients (Benjafield et al., 2019).

Delays in the diagnosis and treatment of OSA can have serious adverse effects on public health and healthcare costs, prompting extensive research in the areas of prevention, diagnosis, and treatment to implement effective measures for early detection and intervention. In recent years, OSA research has emerged as a hot topic in multidisciplinary studies, encompassing fields such as otorhinolaryngology, stomatology, respiratory medicine, neurology, among others. The focus has particularly been on the selection and application of diagnostic methods. Current diagnostic methods for OSA detection include polysomnography, upper airway manometry, imaging tests (upper airway X-ray, CT, MRI examination), electronic fibro-laryngoscope, sleep endoscopy, and acoustic reflexes. Polysomnography is considered the most reliable confirmatory test for OSA diagnosis (Neelapu et al., 2017), providing indications about respiratory status, gas flow, oxygen saturation, and sleep status (Cagle et al., 2023). However, polysomnography monitoring requires subjects to be monitored in the examination room throughout the night, and the test results often require manual data analysis by specialized technicians, requiring high equipment examination environment, examination, and analysis, making it more suitable for confirming the diagnosis of patients with typical symptoms rather than clinical screening. Upper airway manometry is invasive, costly, and inefficient, with poor patient compliance (Sundaram et al., 2005). Imaging tests can only reflect the collapse of the upper airway during the waking phase and are static examinations that cannot be observed dynamically, often having a large gap with the sleep phase (Bommineni et al., 2023; Kim et al., 2023). Sleep endoscopy allows dynamic observation of the collapse of the upper airway during the patient's sleep, facilitating the determination of the site and degree of obstruction more intuitively. However, the examination is complicated, requiring close cooperation and full monitoring by the examining physician and anesthesiologist, and carries certain risks of anesthesia (Kent et al., 2023). Other examination techniques, such as acoustic reflexes, are still in the research stage and not widely carried out in the clinic (Ravesloot and de Vries, 2011). In conclusion, current OSA screening tools have problems such as low examination efficiency, poor patient compliance, and high prices, which leave many patients with OSA undiagnosed in the early to mid-stage and long-term and potentially pathological damage without timely intervention and treatment. Therefore, accurate screening for early and mid-stage patients is essential. The latest guidelines from the American Academy of Sleep Medicine (AASM) also point out that the establishment of a clinical prediction model to screen patients with a high probability of OSA and prioritize PSG examination can benefit patients (Ferreira-Santos et al., 2022).

In recent years, machine learning, as a vital branch of artificial intelligence, has found widespread applications in various fields, including data mining, model building, and image recognition (Choi et al., 2020). Its significant impact on medical processes has garnered increasing attention from medical researchers due to its remarkable effectiveness (Gutiérrez-Tobal et al., 2022). Traditional data analysis methods rely on the perspectives and opinions of analysts, systematically forming fixed patterns. In contrast, machine learning has the capability to continuously and iteratively learn, progressively enhancing model performance (Greener et al., 2022). Moreover, machine learning disease prediction models are particularly suited for early disease screening in large populations, providing early indications for diseases that may require in-depth examinations for a definitive diagnosis, thereby potentially saving significant medical costs. The algorithmic models are not only easy to operate and efficient but also have a broad range of applications. Thus, fully leveraging the advantages of machine learning and combining them with the high-risk factors of clinical diseases and diagnostic methods can play a pivotal role in disease prediction.

Relevant machine learning models have demonstrated success in various diseases such as diabetic retinopathy (Bora et al., 2021), new-onset atrial fibrillation (Raghunath et al., 2021), and lung cancer (Heuvelmans et al., 2021), bringing considerable convenience to clinical screening and contributing to cost savings in medical care. Building upon these observations, given the high incidence of OSA, we aim to leverage machine learning models—an accurate, fast, simple, and cost-effective method—to make preliminary predictions for a large population. The goal is to screen and diagnose as many patients with OSA in the early and middle stages as possible, facilitating timely clinical interventions to reverse the pathological changes caused by OSA and reduce associated pathological burdens. Therefore, this study proposes the utilization of five machine learning algorithms—Extreme Gradient Boosting (XGBoost), Logistic Regression (LR), Support Vector Machine (SVM), Light Gradient Boosting Machine (LightGBM), and Random Forest (RF)—to gather basic information [sex, age, height, weight, and body mass index (BMI)], medical history (clinical symptoms and comorbid diseases), Epworth Sleepiness Scale (ESS), and polysomnography findings. We aimed to construct a machine learning model for the automatic screening and diagnosis of OSA, providing a simple and fast tool for further epidemiological investigations of OSA.

## 2 Methods

### 2.1 Study subjects

A total of 439 patients, including 102 normal patients, 100 patients with mild OSA, 95 patients with moderate OSA, and 142 patients with severe OSA, who underwent polysomnography at the Affiliated Hospital of Xuzhou Medical University from October 2020 to October 2023 were retrospectively selected. The OSA diagnostic criteria were taken from the Clinical Practice Guideline for Diagnostic Testing for Adult Obstructive Sleep Apnea: An American Academy of Sleep Medicine Clinical Practice Guideline (Kapur et al., 2017). Patients with the following conditions were excluded:

1. Acute upper respiratory tract infection.
2. Unstable or decompensated cardiopulmonary disease.
3. Benign and malignant tumors.
4. Serious physical and mental diseases.
5. Recent upper airway surgery and tracheostomy.

The study received approval from the Medical Ethics Committee of the Affiliated Hospital of Xuzhou Medical University (Approval No. XYFY-KL341-01).

### 2.2 Study methods

#### 2.2.1 Polysomnography

Polysomnography is used for continuous and simultaneous acquisition, recording, and analysis of multiple sleep physiological indicators and pathological events during sleep. The Emboletta sleep detection system, model: Embletta X100, was utilized for polysomnography in this study. A designated technician recorded patient information, facilitated the placement of the sleep detector, and undertook the analysis and extraction of data post-examination. Polysomnography serves as a fundamental tool for the analysis of sleep structure and the assessment of sleep disorders, being crucial for clinical and scientific research in sleep medicine (Rundo, 2019).

#### 2.2.2 Grouping criteria

The assessment of sleep apnea severity primarily relies on the apnea-hypopnea index (AHI) (Martinez-Garcia et al., 2023). According to the AHI, the severity of OSA is categorized into three degrees: mild (AHI > 5–15), moderate (AHI > 15–30), and severe (AHI > 30). The AHI is calculated as the sum of the number of apneas and hypoventilation divided by the sleep time, representing sleep breathing disorders [AHI = (number of apneas + hypoventilation)/sleep time (hour)].

#### 2.2.3 Included variables

1) Demographic information: Age, sex, height, weight, and BMI (Cho et al., 2016; Mokhlesi et al., 2016; Senaratna et al., 2017; Lo Bue et al., 2020).
2) ESS: The ESS assesses subjects' tendency to sleepiness during the day based on eight conditions. Each condition is scored on a 0–3 scale for the likelihood of dozing or falling asleep, resulting in a total score of 24. Interpretations include the following:
  - >6: tendency to sleepiness,
  - >11: significant sleepiness,
  - >16: severe drowsiness.
  - Conditions assessed included the following:
  - When sitting and reading,
  - When watching TV,
  - When sitting and not moving in public (such as meetings or theater),
  - When traveling by car for 1 h without interruption,
  - When sitting and talking with others,
  - When sitting quietly after lunch (without drinking alcohol),
  - When lying down in the afternoon to rest,
  - When driving and waiting for the signal,
  - Presence of significant drowsiness suggested when the total score was >10 (Chiu et al., 2017; Gandhi et al., 2021).
3) Disease history: snoring, nocturnal awakening, morning headache, memory and concentration loss, gastroesophageal reflux, and morning dry mouth (yes, no) (Morrell et al., 2003; Stark and Stark, 2015; Patel, 2019; Zhang et al., 2021).
4) Comorbid diseases: Hypertension, coronary heart disease, arrhythmia, thyroid disease, and cerebral cardiovascular disease (yes, no) (Petrone et al., 2016; Strausz et al., 2021; Yeghiazarians et al., 2021; Redline et al., 2023).

#### 2.2.4 Data preparation and cleaning

The initial step was to assess whether the proportion of missing values in each dataset exceeds 95%. If this threshold is surpassed, eliminate the rows and columns containing the missing values to obtain a final dataset devoid of any missing values. For datasets where missing values account for no more than 95%, initiate telephone contact to enhance the information. In instances where contact cannot be established, fill continuous variables with the mean, rank variables with the median, and unordered variables with the mode.

During the data processing phase, textual data is commonly encountered. Algorithms such as LR and SVMs cannot directly handle textual data, necessitating the conversion of textual data into numerical form before use. For instance, {“female,” “male”} and {“hypertension,” “coronary heart disease,” “arrhythmia,” “thyroid disease”} represent aggregated forms of category values. Label encoding, a prevalent method, is used when variables are numerical or exhibit a certain logical relationship. In cases where data are categorical, numbering using consecutive integers in the interval [0, n-1] is adopted. For example, assigning 0 for hypertension, 1 for coronary artery disease, 2 for arrhythmia, and so forth. While this method introduces a degree of continuity to the data, the numbers solely represent categories, and the underlying substance remains non-continuous. To mitigate this issue, One-Hot coding is applied to the preprocessing of category data. This enhances the rationality in similarity and distance calculations and facilitates better application to relevant machine models, thereby enhancing credibility (Qiao et al., 2019).

It is imperative in practical applications to differentiate between data categories and determine whether they belong to ordered or categorical information. For data types with no logical relationship between values, such as “male” and “female,” One-Hot coding is most suitable. In contrast, for ordered data like “mild,” “moderate,” and “severe,” possessing a logical relationship, One-Hot coding is still appropriate, with the numbers representing logical relationships remaining intact throughout the coding process (Jia et al., 2018).

#### 2.2.5 Optimization of parameters

The optimization of the model utilizes the Grid Search CV (cross validation) algorithm, a two-stage process encompassing grid search and cross-validation (Krishnan et al., 2022). Grid search involves traversing multiple parameter combinations, earning its title of exhaustive search. The objective is to identify optimal parameters by iteratively adjusting them and training the learning algorithm with the adjusted parameters. This process continues until all possible parameters are explored, enabling the discovery of the best combination.

The algorithm automatically organizes and combines parameter values when dealing with smaller datasets, effectively enhancing machine learning efficiency. The guiding principle (Borstelmann, 2020) involves initially selecting the parameter with the greatest impact on the model for tuning. Sequential searches within specified value intervals are performed until the optimal value is determined. This process is then repeated for the next parameter with a significant influence, continuing until all parameters are tuned and the best combination is identified.

To mitigate overfitting and underfitting, a grid search method based on K-fold cross-validation is used. This method, combined with K-fold cross-validation, aims to enhance the model's prediction accuracy. The K-fold cross-validation method (Doupe et al., 2019) divides all samples into K parts, using one part as the test set and the rest as the training set in each experiment. Repeating this process K times yields K models. The average of all evaluation indices after K experiments serves as the assessment for parameter tuning. The model's final parameters are determined based on the best evaluation index. Typically, K is set to values like 5 or 10, and in this study, K = 5 is chosen. By comparing values within each set, the parameter yielding the highest prediction accuracy within the selected range can be identified.

#### 2.2.6 Introduction to algorithms

The SMOTE algorithm is a constant for unbalanced data enrichment proposed by Chawla. The basic principle is to perform random linear interpolation between the few class samples and their neighbors to complete the data enrichment to achieve a certain imbalance ratio. The imbalance ratio is the ratio of the number of samples of few classes to the number of samples of multiple classes in the sample set (Wang et al., 2021; Hassanzadeh et al., 2023).

The LR model stands out as a machine learning model known for its simplicity and good interpretability, making it one of the most widely used methods in clinical research. This model is transformed into an excellent classification algorithm by incorporating a sigmoid function onto linear regression. LR is computationally inexpensive, easy to understand, implement, and demands minimal computational resources, making it fast and efficient in classifying tasks. In contrast, the SVM is a robust method for constructing classifiers. It establishes a judgment boundary, referred to as a hyperplane, between two classes. SVM seeks the optimal partitioning of the feature space (normal-abnormal) with this hyperplane by maximizing the margin, which is the distance from the plane to the support vector—the nearest point to the plane (Peng et al., 2023).

RF, characterized by its simple structure and ease of implementation, presents a low computational overhead and proves effective in addressing high-dimensional problems. RF compensates for the limitations of traditional models in handling complex interactions. It also provides valuable information such as important measures of variables, demonstrating advantages in classification accuracy and stable performance (Hu and Szymczak, 2023).

XGBoost is a popular machine learning method utilizing decision trees as the underlying learner for implementing gradient boosting. It iteratively constructs simple regression trees by finding partition values that minimize the prediction error among all input variables. The iterative process involves building additional regression trees with the same structure, where each regression tree minimizes the residuals of the previous ones (Woillard et al., 2021). XGBoost enhances predictions by sequentially building trees, training each tree to address the remaining prediction error after the previous tree. It controls the depth and complexity of individual trees, contributing to the creation of complex and accurate models (Docherty et al., 2021). In comparison, the LightGBM offers advantages such as faster training efficiency, occupying less memory space, achieving higher accuracy rates, and supporting parallelized learning. This method iteratively trains weak classifiers to obtain the optimal model. Additionally, it employs a Leaf-wise leaf growth strategy based on depth limitation. In each iteration, it identifies the leaf node with the largest splitting gain from all current leaf nodes and splits it, thereby reducing errors (Park et al., 2021). Two techniques, GOSS and EFB, enhance traditional gradient boosting iterative decision trees. The GOSS algorithm saves samples with larger gradients, while the EFB algorithm bundles a large number of exclusive features onto much less dense features. This combination, along with the GOSS and EFB algorithms, efficiently handles large-scale data samples and feature extraction, preventing unnecessary computation of zero eigenvalues (Deng et al., 2018; Zhan et al., 2018).

#### 2.2.7 Evaluation metrics of the model

To evaluate the method proposed in this study for comparison with other methods, four commonly used metrics were used, including **F1 score**; **specificity** (true negative rate, **TNR**), TNR = TN/(FP + TN); **sensitivity** (true positive rate, **TPR**), TPR = TP/(TP+ FN); **accuracy** (**ACC**), **ACC** = TP+ TN/(TP + FP + TN + FN); area under the receiver operating characteristic curve **(ROC) (AUC)** (Chatterjee et al., 2020; Namkung, 2020).


$$\begin{array}{r} \text{Accuracy}=\frac{\text{TP}+\text{TN}}{\text{TP}+\text{FP}+\text{TN}+\text{FN}}\\ \text{TPR}=\frac{\text{TP}}{\text{TP}+\text{FN}}\\ \text{TNR}=\frac{\text{TN}}{\text{TN}+\text{FP}} \end{array}$$

TP refers to the number of correctly classified positive samples, FP refers to the number of misclassified negative samples, TN refers to the number of correctly classified negative samples, and FN refers to the number of misclassified positive samples. Since the two types of samples in the dataset were not uniformly distributed, the overall performance could be well evaluated by using accuracy, sensitivity, and specificity metrics; thus, we focused on AUC, which is usually between 0.5 and 1 and is an important reference to evaluate the model fitting effect. When the AUC value of the model is ≥0.7, the model fits better; when the AUC is ≥0.9, the model has a very strong predictive power and its performance is better. Furthermore, the closer the AUC value of the model is to 1, the better the performance. The larger the AUC, the better the performance of the model.

#### 2.2.8 Modeling process

**Figure 1:**
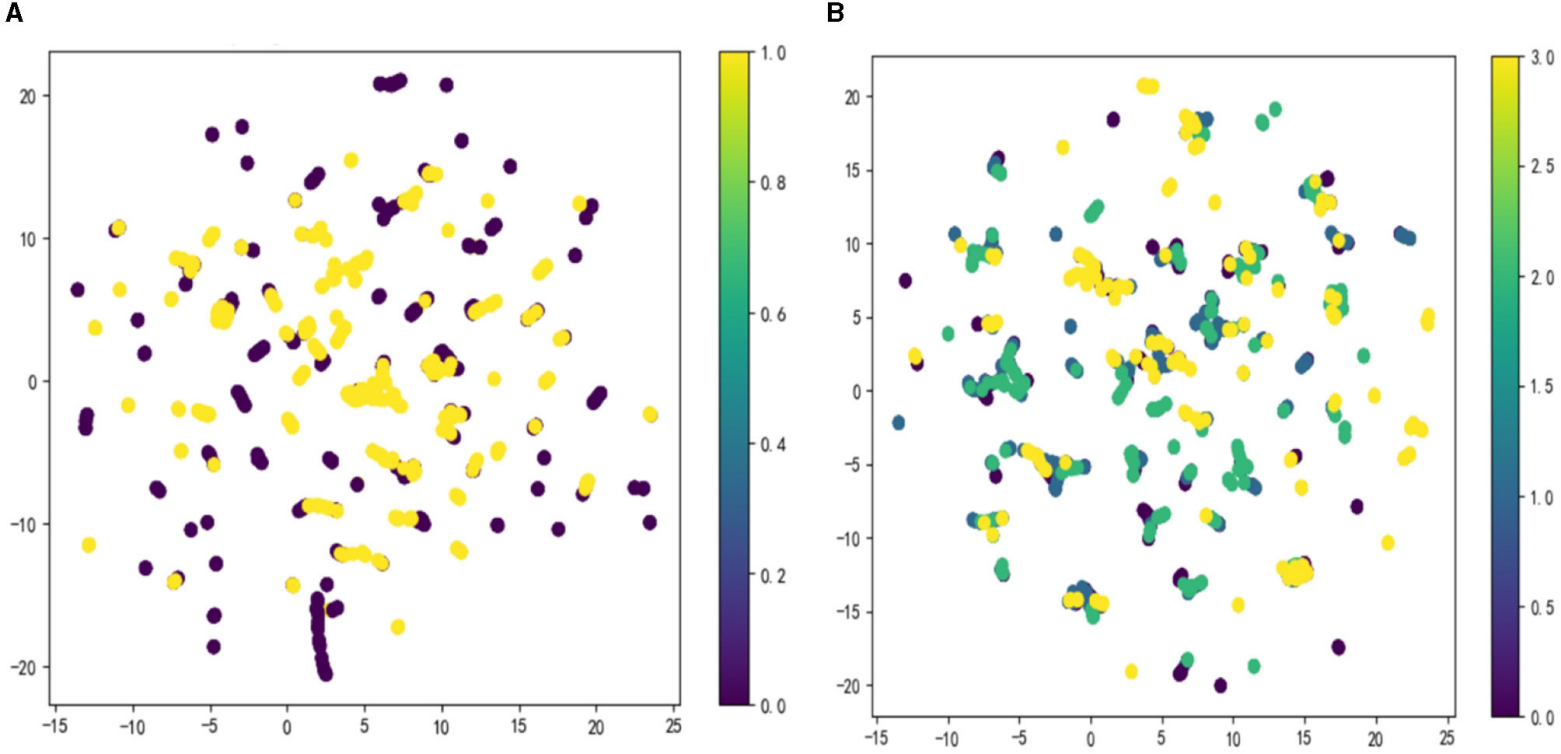
Visualize the data using the Umap algorithm: **(A)** binary classification; **(B)** multi-categorization.

**Figure 2:**
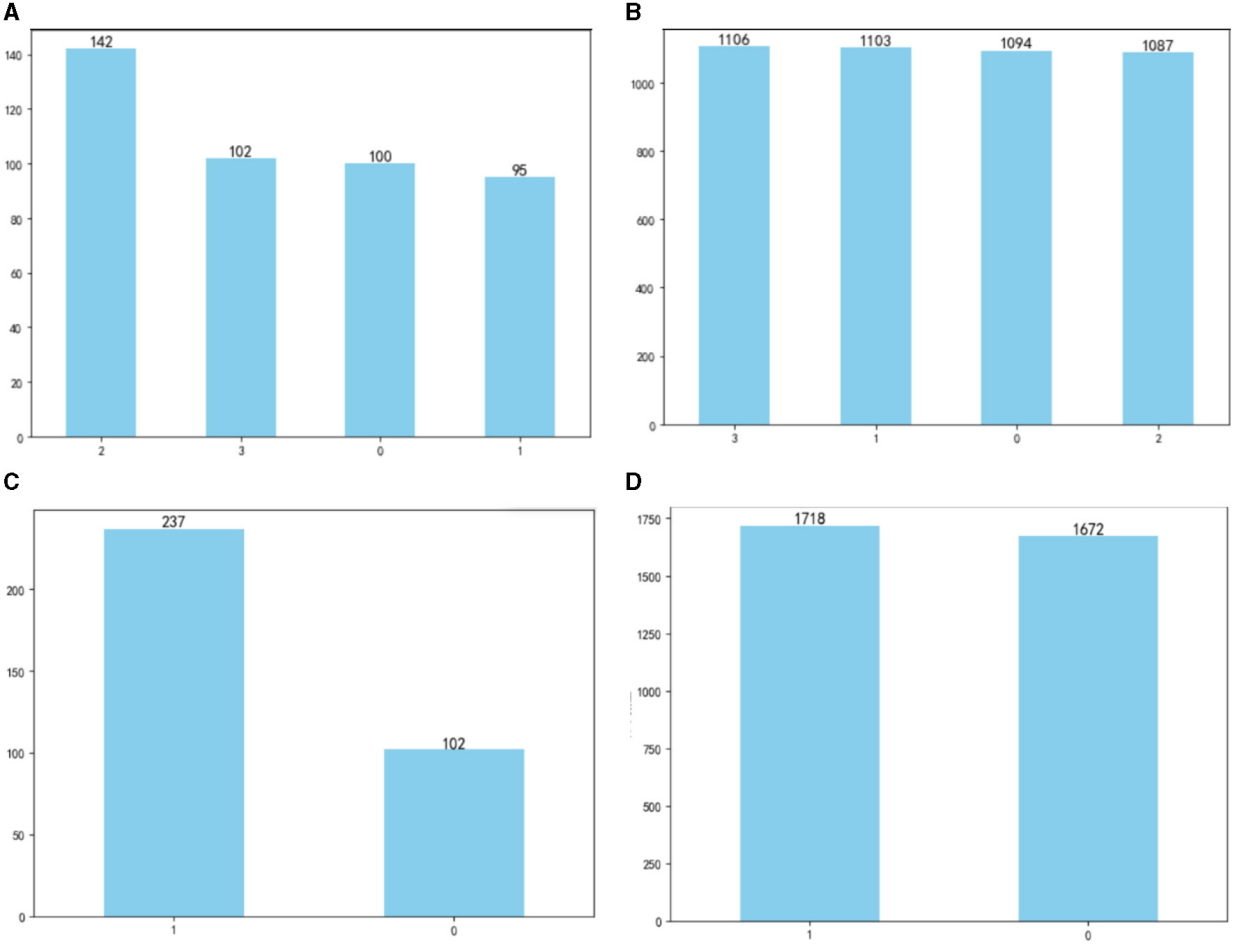
Comparison of data before and after amplification: **(A)** before amplification; **(B)** after amplification; **(C)** before amplification; **(D)** after amplification.

The OSA prediction model utilized the LightGBM algorithm, LR algorithm, XGBoost algorithm, RF algorithm, and SVM algorithm. Firstly, we use the Umap algorithm to map the data into 2D and visualize the data differently using the target variable as a color (Figure 1). Secondly, the SMOTE algorithm is used to amplify the original data, which is 10 times that of the original data, and the amplified data is in a balanced state (Figure 2). Then dataset was split into an 80% training set and a 20% test set. Predictor variables were input into each of the five algorithms to build the respective models. Subsequently, the remaining 20% of patients served as the test set, with predictor variables input into the models for quadratic and dichotomous calculations. The output results were then compared with the polysomnography results to assess the accuracy of the models.

#### 2.2.9 Statistical methods

Statistical analysis of the data was conducted using SPSS 25.0. The normality of measurement data was tested using the Shapiro–Wilk test. For measurement data conforming to normal distribution, mean ± standard deviation was used, and the independent samples *t*-test facilitated group comparisons. Skewed distribution measures were expressed as median (quartiles), and the Mann–Whitney U and Kruskal–Wallis rank sum tests were used for dichotomous and quadratic group comparisons, respectively. Count data were presented as the number of cases, with group differences assessed using the chi-squared (χ^2^) test. A significance level of P < 0.05 was considered statistically significant.

The prediction model was developed using LightGBM in Python with the Scikit-learn package. For two types of models, 80% out of patients were randomly chosen in a 4:1 ratio as the training set, while the remaining 20% served as the test set.

Five models, namely LR, SVM, XGBoost, LightGBM, and RF, were constructed using the training set. Model reliability was assessed using the test set, with the AUC chosen as the evaluation metric. A larger AUC indicated better predictive model performance.

## 3 Results

### 3.1 Four classification models

#### 3.1.1 Comparison between groups of each basic variable of the four classifications

In the cohort of 439 patients across the four classification models, the distribution was as follows: 1,096 classified as normal, 1,117 as mild, 1,090 as moderate, and 1,087 as severe. Significant differences (*P* < 0.05) were observed among the four groups in terms of the following basic variables: sex, age, BMI, significant snoring, nocturnal awakening, memory and attention impairment, gastroesophageal reflux, morning dry mouth, total ESS score, Coronary heart disease, Arrhythmia, Thyroid disease, Cerebrovascular disease and hypertension (Table 1).

**Table 1:** Comparison of various parameters between groups with different degrees of OSA (four categories).

**Parameters**	**Normal (*n* = 1,096)**	**Mild (*n* = 1,117)**	**Moderate (*n* = 1,090)**	**Severe (*n* = 1,087)**	***P***
Male	812	1,029	959	1,048	< 0.001
Age	37 (29, 49)	41 (32, 51)	45 (34, 53)	42 (33, 53)	< 0.001
BMI	23.51 (22.05, 25.74)	25.34 (23.75, 27.04)	25.44 (24.16, 26.97)	28.73 (26.50, 30.39)	< 0.001
Snoring	995	1,113	1,087	1,084	< 0.001
Waking up at night with suffocation	523	761	825	806	< 0.001
Morning headache	227	409	395	397	< 0.001
Loss of memory and concentration	508	966	869	887	< 0.001
Gastroesophageal reflux	160	576	363	590	< 0.001
Dry mouth	410	1,010	1,025	998	< 0.001
ESS total score >10	0	165	213	463	< 0.001
Hypertension	177	404	275	577	< 0.001
Coronary heart disease	85	62	44	143	< 0.001
Arrhythmia	135	159	57	113	< 0.001
Thyroid disease	112	135	155	39	< 0.001
Cerebrovascular disease	46	104	213	158	< 0.001

#### 3.1.2 Parameters of five prediction models for four classifications

In the four-classification model, the data comparison between the training set and the test set and the selection of the optimal parameters by the grid search algorithm are shown in the Tables 2, 3.

**Table 2:** Training and validation (four categories).

**Parameters**	**Training (*n* = 3, 512)**	**Validation (*n* = 878)**	***P***
Male	3,079	769	0.896
Age	40 (32, 58)	41 (32, 58)	0.515
BMI	25.84 (23.65, 27.72)	25.68 (23.62, 29.81)	0.261
Snoring	3,416	863	0.084
Waking up at night with suffocation	2,339	576	0.576
Morning headache	1,137	291	0.664
Loss of memory and concentration	2,560	670	0.040
Gastroesophageal reflux	1,352	337	0.951
Dry mouth	2,979	740	0.690
ESS total score	675	163	0.659
Hypertension	1,158	275	0.351
Coronary heart disease	271	63	0.589
Arrhythmia	372	92	0.922
Thyroid disease	363	88	0.785
Cerebrovascular disease	419	102	0.797

**Table 3:** Parameters of each model of the four classifications.

**Model**	**Parameters**
LR	‘C': 0.1
SVM	‘C': 0.1, ‘gamma': 0.1, ‘kernel': ‘poly'
RF	‘max_depth': 7, ‘n_estimators': 100
XGBoost	‘learning_rate': 0.1, ‘max_depth': 3, ‘n_estimators': 50
LightGBM	‘learning_rate': 0.1, ‘max_depth': 3, ‘n_estimators': 150

#### 3.1.3 Comparison of evaluation metrics of the five models

For the four classification models, utilizing 20% (88 cases) as the test set for validation, the AUC ranking of the five algorithms is as follows: LightGBM > RF SVM > XGBoost > LR. The accuracy ranking is LightGBM > RF > SVM > XGBoost > LR. LightGBM exhibiting the highest sensitivity among them. The specificity ranking was as follows: LightGBM > RF > SVM = XGBoost > LR.

**Figure 3:**
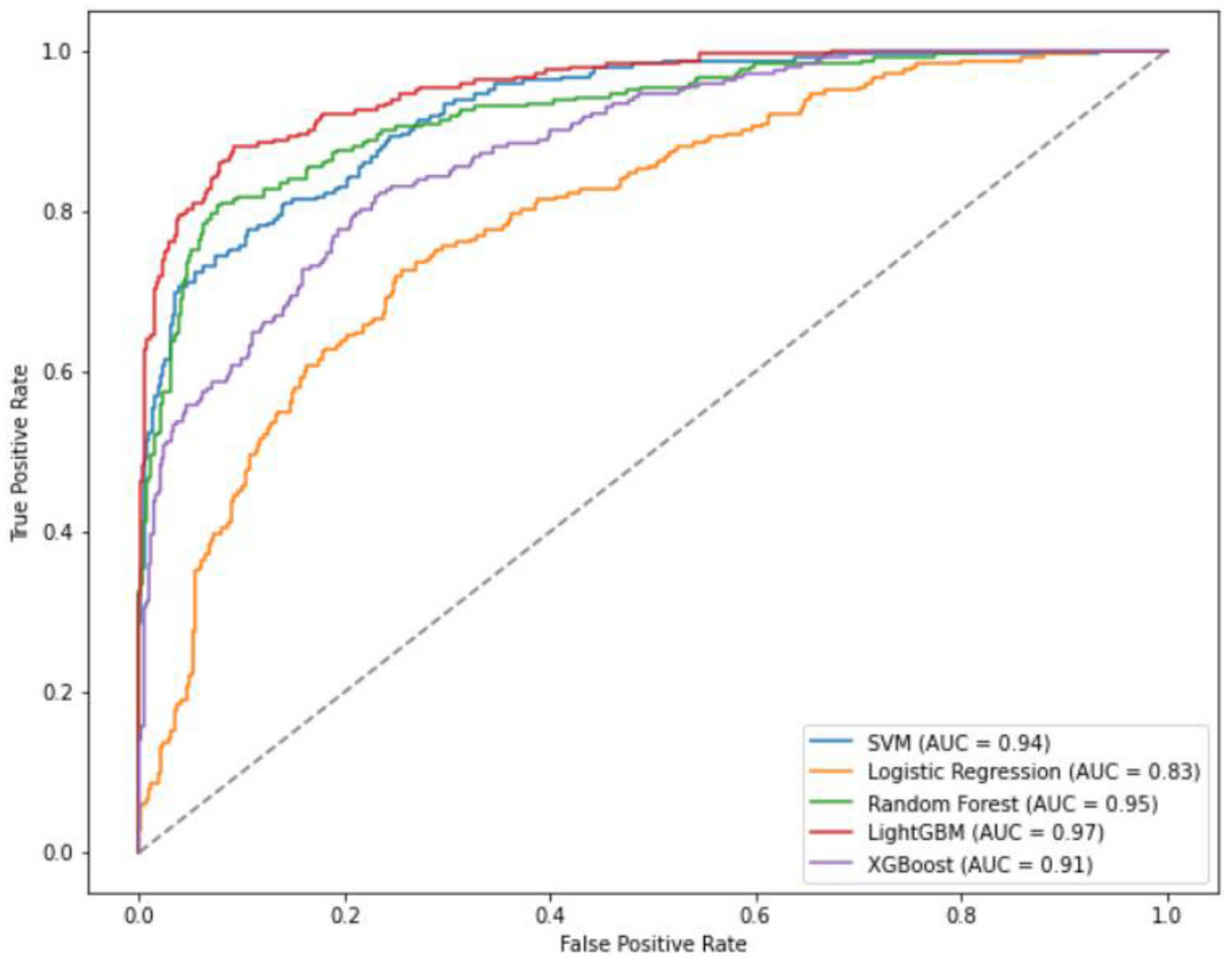
ROC curves of four classification and five models.

Specifically, the accuracy of LightGBM was 0.93 (AUC = 0.97); LR achieved an accuracy of 0.71 (AUC = 0.83); XGBoost attained an accuracy of 0.81 (AUC = 0.91); SVM showed an accuracy of 0.84 (AUC = 0.94); RF achieved an accuracy of 0.88 (AUC = 0.95) (Table 4, Figure 3). Across the four classifications, the AUC values for all five models were higher than 80%, with LightGBM outperforming LR, XGBoost, RF, and SVM in each evaluation index.

**Table 4:** Comparison of prediction performance of five models with four classifications.

**Evaluation indicators**	**LightGBM**	**XGBoost**	**SVM**	**LR**	**RF**
AUC	**0.97**	0.91	0.94	0.83	0.95
Accuracy	**0.93**	0.81	0.84	0.71	0.88
Sensitivity	**0.96**	0.85	0.92	0.72	0.94
Specificity	**0.91**	0.78	0.78	0.70	0.84
F1	**0.93**	0.79	0.84	0.68	0.88

The meaning of the bold values is to highlight the evaluation indicators of the optimal model.

#### 3.1.4 Importance ranking of the variables

**Figure 4:**
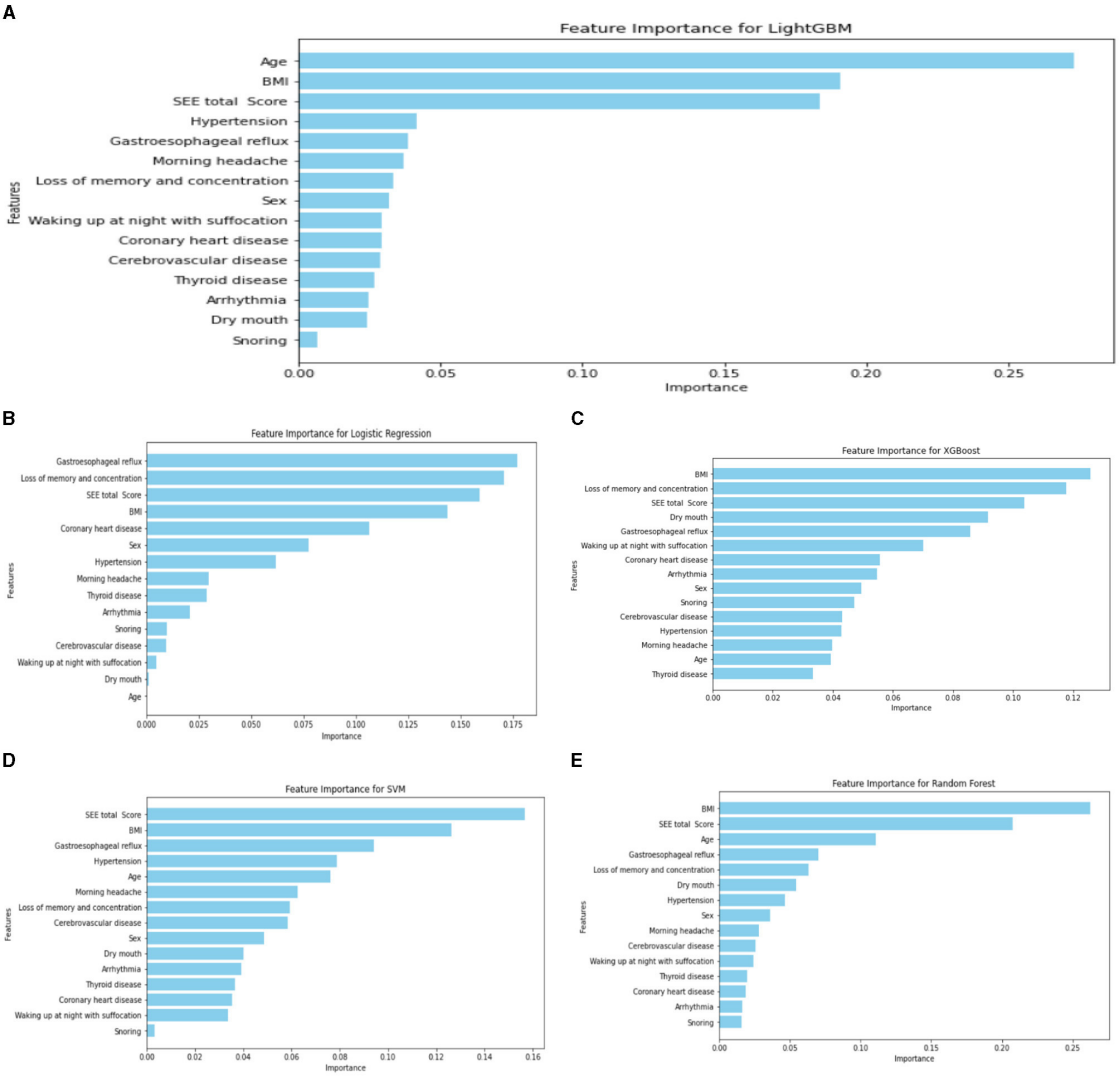
Ranking chart of the importance of variables of five algorithms in the four-classification OSA classification model. **(A)** LightGBM; **(B)** LR; **(C)** XGboost; **(D)** SVM; **(E)** RF.

Regarding the importance rankings of statistically different variables, there were variations in the feature importance rankings among RF, LR, XGBoost, RF, and SVM. In particular, the top five feature importance rankings for LightGBM, which exhibited the best evaluation index, were Age, BMI, ESS total score, hypertension, and gastroesophageal reflux (refer to Figure 4). Notably, Age, ESS total score and BMI stood out as the most prominent factors in determining importance.

### 3.2 Binary classification model

#### 3.2.1 Comparison of basic variables in dichotomous classification

Within the 3,390 patients in the dichotomous model, comprising 1,718 individuals in the moderate-to-severe OSA group and 1,672 in the normal group, notable differences were identified between the two groups. These distinctions encompassed sex, age, BMI, significant snoring, nocturnal awakening, memory and attention loss, gastroesophageal reflux, morning dry mouth, total ESS score, hypertension, and cerebrovascular disease (*P* < 0.05) (refer to Table 5).

**Table 5:** Comparison of parameters between the non-OSA group and the moderate-to-severe OSA group (dichotomous classification).

**Parameters**	**Non-OSA (*n* = 1,672)**	**OSA (*n* = 1,718)**	**P**
Male	1,243	1,594	< 0.001
Age	36 (30, 49)	43 (34, 53)	< 0.001
BMI	23.53 (22.16, 25.69)	27.18 (25.38, 29.61)	< 0.001
Snoring	1,538	1,712	< 0,001
Waking up at night with suffocation	814	1,224	< 0.001
Morning headache	348	592	0.067
Loss of memory and concentration	760	1,358	< 0.001
Gastroesophageal reflux	231	803	< 0.001
Dry mouth	1,052	1,595	< 0.001
ESS total score	0	567	< 0.001
Hypertension	294	770	< 0.001
Coronary heart disease	128	153	0.649
Arrhythmia	207	157	0.509
Thyroid disease	168	111	0.631
Cerebrovascular disease	84	256	0.025

#### 3.2.2 Parameters of five prediction models for dichotomous classification

In the binary classification model, the data comparison between the training set and the test set and the grid search algorithm are used to select the most important parameters in the Tables 6, 7.

**Table 6:** Training and validation (dichotomous classification).

**Parameters**	**Training (*n* = 2,712)**	**Validation (*n* = 678)**	***P***
Male	2,279	558	0.275
Age	40 (32, 51)	41 (32, 51)	0.515
BMI	25.71 (23.70, 27.73)	25.68 (23.62, 27.45)	0.261
Snoring	2,597	653	0.517
Waking up at night with suffocation	1,632	406	0.888
Morning headache	758	182	0.565
Loss of memory and concentration	1,711	407	0.141
Gastroesophageal reflux	850	184	0.033
Dry mouth	2,129	518	0.237
ESS total score	453	114	0.945
Hypertension	846	218	0.630
Coronary heart disease	219	62	0.366
Arrhythmia	301	63	0.174
Thyroid disease	226	53	0.662
Cerebrovascular disease	274	66	0.775

**Table 7:** Parameters of each model of binary classification.

**Model**	**Parameters**
LR	‘C': 0.1
SVM	‘C': 1, ‘gamma': 0.01, ‘kernel': ‘rbf'
RF	‘max_depth': 6, ‘n_estimators': 70
XGBoost	‘learning_rate': 0.02, ‘max_depth': 2, ‘n_estimators': 50
LightGBM	‘learning_rate': 0.01, ‘max_depth': 2, ‘n_estimators': 50

#### 3.2.3 Comparison of evaluation indicators of five models

In the binary classification model, utilizing 20% of the dataset (678 cases) as the test set for validation, the AUC rankings of the five algorithms are as follows: RF > LightGBM = LR > XGBoost > SVM. The accuracy ranking is RF > SVM > LR > XGBoost > LightGBM. For sensitivity, the ranking is RF = SVM > LR > XGBoost > LightGBM, and for specificity, it is RF > XGBoost = SVM > LightGBM > LR.

**Figure 5:**
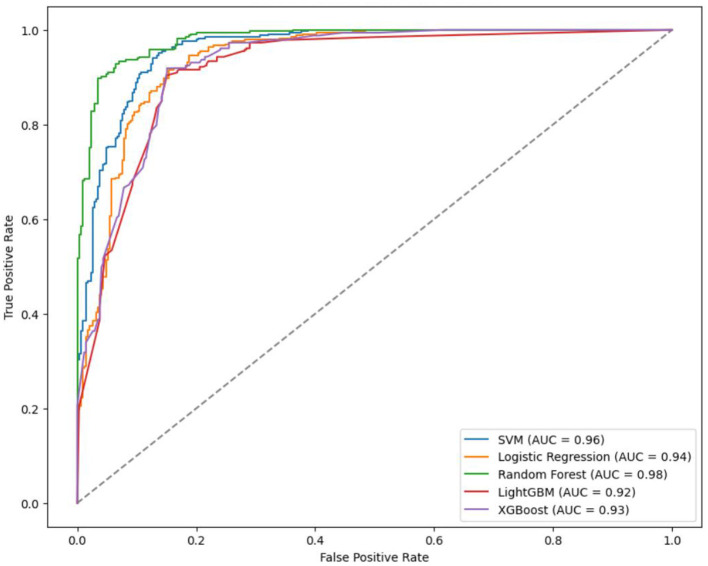
ROC curves of the five models of binary classification.

The prediction accuracy of LightGBM was 0.85 with an AUC of 0.92, LR achieved an accuracy of 0.88 and an AUC of 0.94, XGBoost had a prediction accuracy of 0.86 with an AUC of 0.93, SVM showed an accuracy and AUC of 0.90 and 0.96. respectively, and RF outperformed with an accuracy and AUC of 0.91 and 0.96 respectively (Table 8, Figure 5). In the binary classification model, all five algorithms exhibit AUC values exceeding 90%, indicating high predictive performance. RF stands out with superior AUC, accuracy, sensitivity, and specificity than LR, XGBoost, LightGBM, and SVM.

**Table 8:** Comparison of prediction performance of five binary classification models.

**Evaluation indicators**	**LightGBM**	**XGBoost**	**SVM**	**LR**	**RF**
AUC	0.92	0.93	0.96	0.94	**0.98**
Accuracy	0.85	0.86	0.90	0.88	**0.91**
Sensitivity	0.77	0.79	0.87	0.84	**0.87**
Specificity	0.93	0.94	0.94	0.92	**0.96**
F1	0.84	0.85	0.90	0.87	**0.91**

The meaning of the bold values is to highlight the evaluation indicators of the optimal model.

#### 3.2.4 Ranking of the importance of each variable

**Figure 6:**
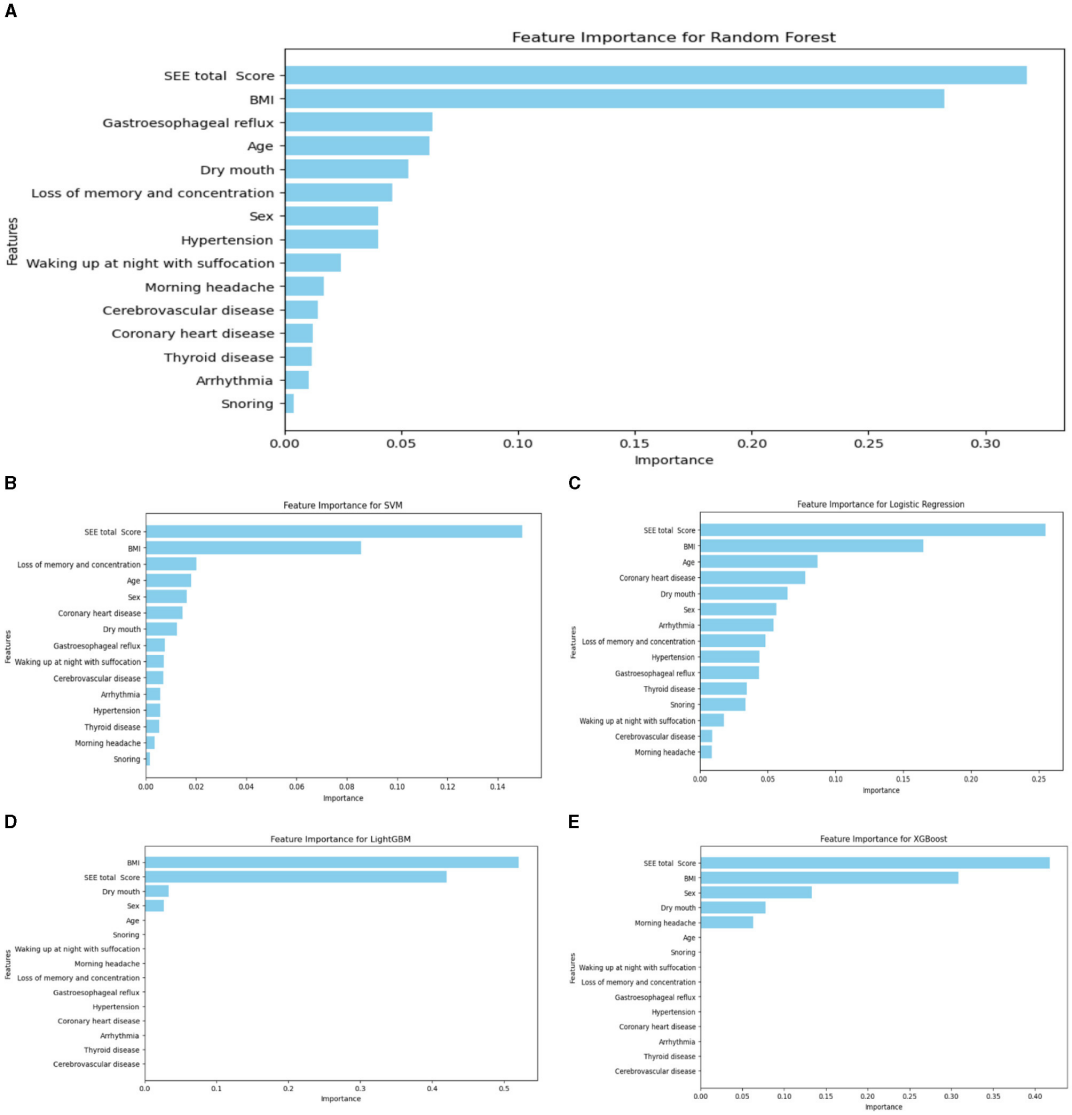
Ranking chart of the importance of variables of five algorithms of the binary classification OSA screening model. **(A)** RF; **(B)** SVM; **(C)** LR; **(D)** LightGBM; **(E)** XGboost.

**Figure 7:**
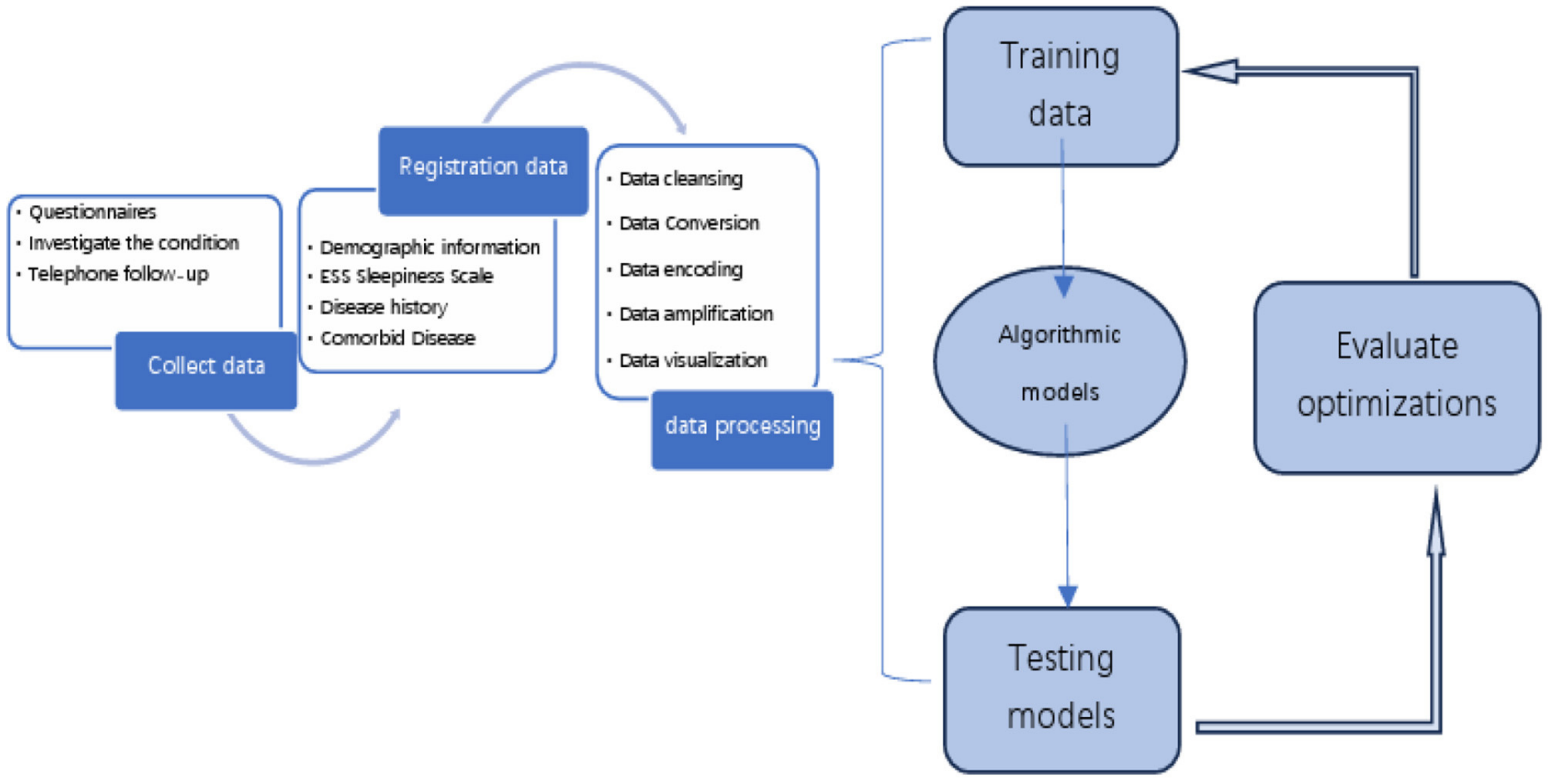
Overall framework flowchart.

The importance rankings of variables such as sex, age, BMI, significant snoring, nocturnal awakening, memory and attention loss, gastroesophageal reflux, dry mouth in the morning, drowsiness, hypertension, and cerebrovascular diseases varied among LightGBM, LR, XGBoost algorithm, RF, and SVM. The top five feature importance rankings for RF, which exhibited the highest evaluation indicators, were ESS total score, BMI, age, gastroesophageal reflux, and sex (Figure 6). Notably, the total ESS score and BMI emerged as particularly crucial variables in the predictive performance of the model. The overall flow chart of this study is shown in the figure below (Figure 7).

## 4 Discussion

Polysomnography is widely recognized as the gold standard for diagnosing OSA, yet its use for OSA screening is hindered by operational complexities, time requirements, and economic costs. In response to these limitations, various life scales, such as the ESS, Quebec sleep questionnaire, and the OSA-18 scale for children, have been used in clinical settings to complement polysomnography. The ESS, comprising subjective sleepiness questions in eight conditions, is widely used despite its subjective nature, as its reliability and validity have been established (Pitre et al., 2023). However, the advent of computer machine learning models has revolutionized disease prediction by integrating and analyzing both subjective and objective indicators. While these models have shown success in predicting diseases like diabetic retinopathy (Bora et al., 2021), new-onset atrial fibrillation (Raghunath et al., 2021), and lung cancer (Heuvelmans et al., 2021), there is a scarcity of studies focusing on machine learning for generating OSA risk prediction models.

Our study aimed to leverage machine learning prediction models to identify patients with OSA at an early stage. Consequently, the selected factors needed to be accurate, fast, simple, and easily interpretable without necessitating extensive ancillary tests. We collated patient demographic information, medical history, ESS, and other subjective and objective indicators. Employing five algorithms—LightGBM, LR, XGBoost, SVM, and RF—we compared the AUC, accuracy, sensitivity, and specificity of these models to establish an automatic screening and diagnostic machine learning prediction model for patients with OSA.

Our study aimed to employ machine learning models for accurate screening of early to mid-stage patients. In the four classifications, to identify models more suitable for the early screening of patients with mild-to-moderate OSA, we categorized the study population into four groups based on AHI: normal, mild, moderate, and severe. Among the five quadruple classification grading models, the LightGBM model demonstrated the best performance (AUC = 0.97, accuracy = 0.93, sensitivity = 0.96, and specificity = 0.91). The AUC of LightGBM outperformed RF, SVM, LR, and XGBoost, with all other indices also showing relatively favorable results. This superiority aligns with findings in other pharmaceutical fields, where Zhang et al. (2019) concluded that LightGBM outperformed SVM and XGBoost in predicting various toxicity or activity-related endpoints for large compound libraries in the pharmaceutical and chemical industries. The top five importance rankings of each variable in the LightGBM model were Age, BMI, ESS total score, hypertension, and gastroesophageal reflux, with the Age, ESS total score and BMI being the most prominent. This alignment with the clinical characteristics of OSA reinforces the relevance of these variables.

In the context of the four classifications, various scholars have reported diverse results, albeit with less-than-satisfactory outcomes. For instance, Mencar et al. (2020) utilized data encompassing demographic characteristics, spirometry values, gas exchange parameters (PaO2, PaCO2), and symptoms (ESS and snoring) from 313 patients with OSA. They established SVM and RF OSA grading prediction models, achieving low performance with an AUC of 65, a sensitivity of 44.7, and an accuracy of 39.9 for SVM and an AUC of 63.7, a sensitivity of 44.1, and an accuracy of 44.1 for RF. Similarly, Bozkurt et al. (2017) employed clinical data, including age, sex, BMI, neck circumference, smoking status, clinical symptoms, and physical examination, to build LR and RF four-category classification prediction models. The AUC for LR and RF was reported as 0.84 and 0.81, respectively, slightly outperforming the models established in this study. This could be attributed to the inclusion of physical examination parameters in their study, leading to more comprehensive and fuller raw data. Physical examinations such as neck circumference, waist circumference, tonsil size, and tongue size play a crucial role in OSA diagnosis (Lim et al., 2014; Wysocki et al., 2016). Future studies might enhance the accuracy of four-category model predictions by incorporating simple and readily available physical examination indices like waist circumference, neck circumference, pharyngeal cavity, heart rate, and blood pressure.

In the dichotomous classification model, each machine-learning model exhibited excellent screening efficacy. This model, focusing on the presence or absence of OSA, surpassed the multi-classification model in terms of performance. Many current disease prediction models emphasize this binary classification due to its superior performance. The gold standard for OSA diagnosis is the polysomnography examination, where the AHI is a crucial indicator of disease severity. However, diagnosing patients with mild OSA remains controversial due to various physiological state changes, such as fatigue, sleeping position, upper airway inflammation, and external stimulants like tobacco and alcohol (Coelho et al., 2022). Patients with moderate-to-severe OSA, on the other hand, have more consistent diagnoses. Zerah-Lancner et al. (2000) showed that AHI is a reliable parameter for estimating OSA severity, particularly with a sensitivity of 100% for AHI ≥ 15, indicating high sensitivity in subjects with moderate or severe disease. Patients with moderate-to-severe OSA experience more severe clinical manifestations, such as sleep fragmentation, producing physical symptoms such as drowsiness and fatigue, and psychological symptoms such as stress, and organ damage compared with patients with mild OSA (Santos et al., 2017; Yan et al., 2022). The association with cardiovascular morbidity is more pronounced in moderate-to-severe cases, emphasizing the need to screen patients with suspected moderate or severe OSA for further diagnosis (Gottlieb and Punjabi, 2020; Sánchez-de-la-Torre et al., 2023). The necessity to screen patients with suspected moderate or severe OSA for further diagnostic confirmation is crucial. Therefore, this study employed a dichotomous classification approach, categorizing normal populations as one group and patients with moderate-to-severe OSA as another group. This strategy aimed to enhance the prediction model's effectiveness in screening for moderate and severe OSA. In the dichotomous LR model, AUC was 0.94, indicating a high prediction accuracy of OSA (88%). This underscores the effectiveness of the LR model in analyzing indicators, with sex being identified as the most significant factor. Comparisons with previous studies reveal noteworthy findings. Zou et al. (2013) achieved an AUC of 0.96 using the LR model with the ESS scale, anthropometric parameters, and lowest oxygen saturation. Saaresranta et al. (2016) developed a screening model with 93% sensitivity but low specificity, leading to a high number of false positives. Xu et al. (2019), using the LASSO approach, attained a lower efficacy (0.75 and 0.78 AUC for predicting moderate and severe OSA). Notably, our study's incorporation of both anthropometric variables and clinical features, including medical history, likely contributed to the heightened predictive power of our model. In the dichotomous LR model, the ranked importance of each variable's characteristics was as follows: drowsiness, BMI, age, coronary heart disease and morning dry mouth. This highlights the relevance of these factors in predicting OSA.

The dichotomous SVM model demonstrated an AUC of 0.96, all indicators were better than LR. In comparison to previous studies, Liu et al. (2017) utilized anthropometric characteristics by SVM, achieving a prediction rate with an AUC of 0.85 for OSA severity. Sharma and Sharma (2016) applied the SVM algorithm to detect sleep apnea based on single-lead electrocardiogram signals, reporting superior results with an AUC of 0.97, a sensitivity of 0.95, a specificity of 1.00, and an accuracy of 0.97. However, their method's limitation was the need for an operator with expertise in electrocardiogram signal extraction, making evaluation challenging. Contrasting conclusions were drawn by Manoochehri et al. (2018), who used age, neck circumference, ESS score, snoring, and other risk factors to establish an SVM vs. LR model for obstructive sleep apnea diagnosis. They found SVM to be superior to LR, with an accuracy of 0.79, a sensitivity of 0.71, and a specificity of 0.84, all lower than the present study. Notably, the top three SVM importance rankings for dichotomous categories were consistent with LR and in the same order: ESS score, BMI and age, suggesting the consistent performance of these variables across LR and SVM models.

In this study, the RF model exhibited superior performance in dichotomous classification, achieving an impressive AUC of 0.98 and an accuracy of 0.91. This aligns with findings from Hajipour et al. (2020), who compared LR and RF models using acoustic features, sex, weight, BMI, and neck circumference. Despite regularization efforts to enhance LR's generalization and prevent overfitting, RF still outperformed regularized LR in accuracy, specificity, and sensitivity by 3.5%, 2.4%, and 3.7%, respectively. The study suggests that when dealing with large datasets requiring rapid real-time screening, regularized LR is a preferable choice due to its relatively fast and accurate classification results. Contrastingly, Wu et al. (2022) utilized hypoxia-related genes and biomarkers to construct an RF model for OSA diagnosis, yielding a lower AUC of 0.667. Notably, their OSA samples exclusively comprised obese cases, a crucial factor in differentiating OSA. This might explain the disparity between their RF model and the current study. Tsai et al. (2022) employed waist circumference, neck circumference, BMI, and visceral fat level to establish risk models for predicting moderate to severe OSA using LR, k-nearest neighbor, Bayesian, RF, SVM, and XGBoost. The RF model, applied to the moderate-severe category, demonstrated an accuracy of 84.74% and an AUC of 90.41%, highlighting its robust performance. This aligns with the current study, where the RF model stood out with an AUC of 0.98 and an accuracy of 0.91 among the dichotomous models. As the top-performing model among dichotomous models, the RF model emphasized the importance of objective variables. The top five variables—drowsiness, BMI, gastroesophageal reflux, age, and morning dry mouth—include two highly objective factors (age and BMI) compared to LR and SVM, underscoring the significance of objective variables in the prediction model. The XGBoost model in dichotomous classification exhibited a robust performance with an AUC of 0.93, an accuracy of 0.86, a sensitivity of 0.79, and a specificity of 0.94. While it ranked as the less accurate and sensitive among the five models in this study, its higher AUC and specificity make it a promising candidate for future investigations. The study by Ye et al. (2023) included involving 3,139 children with suspected OSA used age, sex, BMI, hypoxia index, mean nocturnal heart rate, and fastest heart rate as predictive features for diagnosing mild, moderate, and severe OSA. XGBoost demonstrated AUCs of 0.95, 0.88, and 0.88, with classification accuracies of 90.45%, 85.67%, and 89.81%, respectively. This study regarded XGBoost as a superior performer. Conversely, Kim et al. (2021) utilized clinical symptoms and anthropometric variables, concluding that XGBoost had the lowest OSA prediction performance with sensitivity, specificity, and AUC of 78.69%, 73.91%, and 0.80, respectively. Inconsistencies among these findings may stem from variations in variables, populations, and regions chosen by each study, introducing inevitable biases into the data.

In the dichotomous model, most of the variables in the XGBoost and LightGBM models were underutilized by the models, which was inconsistent with most clinical experience. The most crucial variable in the XGBoost model in dichotomous classification was the total ESS score, reflecting drowsiness and aligning with the clinical manifestations of OSA. The LightGBM model, despite being less utilized in OSA studies, demonstrated exceptional performance just in quadruple classifications. In the dichotomous study, LightGBM achieved an impressive AUC of 0.92. Additionally, in the quadruple classifications, LightGBM outperformed other algorithms, emphasizing its superiority in OSA screening and diagnosis. Given these findings, further exploration and research on LightGBM are warranted to harness its potential in OSA prediction models. A study by Shi et al. (2022) focused on predicting OSA-related hypertension using risk prediction models, including LR, LightGBM, XGBoost, AdaBoost, Bagging, and multilayer perceptron. LightGBM emerged as the top performer with an AUC of 0.885 and accuracy of 0.713. Notably, three variables—BMI, dry mouth, and sleepiness—were identified as significantly more important in LightGBM's dichotomous model.

In both dichotomous and quadruple classifications, the top five characteristics of importance encompassed three indicators: BMI, age, and total ESS score. This consistent ranking underscores the pivotal role these indicators play in influencing OSA predictive outcomes. Drowsiness, reflected in the total ESS score, emerged as the most crucial characteristic parameter for predicting OSA, suggesting its significance as a primary risk factor for differentiating moderate-to-severe OSA. Although only a subset of OSA patients reports excessive sleepiness (Gottlieb et al., 2010), its high prevalence among severe OSA (Lee et al., 2020), cases underscores its importance in clinical assessment (Saaresranta et al., 2016). BMI ranked second in importance, highlighting obesity as a significant risk factor for OSA. Obesity contributes to reduced lung volume, pharyngeal diameter reduction, and fatty deposits in the pharyngeal wall, all contributing to airway narrowing (Carneiro-Barrera et al., 2022). Age also featured prominently as important parameters, with older populations and males being more prone to OSA. The correlation between age and OSA (Liu et al., 2022) is attributed to relaxed nasopharyngeal muscles and increased susceptibility to sleep apnea hypoventilation syndrome with age.

The study developed and validated machine learning models for predicting OSA severity using a four-category OSA graded prediction model and a two-category OSA screening prediction model. LightGBM demonstrated the best performance in the graded prediction model with an AUC of 0.97, indicating some grading ability. However, its clinical application and scalability need further exploration. In screening prediction models, all five algorithms, especially RF, performed well in predicting patients with suspected OSA, with RF achieving the highest AUC of 0.98. This suggests potential for large-scale implementation in clinical and community settings. Comparative analysis between dichotomous and quadruple classification models showed higher metrics in dichotomous classification, emphasizing its effectiveness in predicting OSA.

Limited studies exist on predicting OSA severity (Eiseman et al., 2012), and further research is needed in diverse settings. Machine learning is expected to play a key role in developing clinically useful digital healthcare for OSA and other sleep disorders. The study is based on a retrospective analysis of clinical data, which may introduce biases or limitations in data collection and interpretation. The evaluation of the machine learning models may be influenced by the specific metrics chosen, and additional validation on external datasets could provide further insights into the model's performance and the next step will be additional validation in a wider range of people. The dataset relied more on subjective data, such as the ESS rating scale and medical history, limiting the inclusion of objective parameters. Future research should aim to balance subjective and objective data. Including objective indicators like nasal and pharyngeal cavities, heart rate, blood pressure, and physical examination results will enhance the comprehensiveness of the OSA dataset. The sample size in the study is considered insufficient, affecting the generalizability of model predictions. Conducting studies with larger and diverse samples will validate the generalizability of the developed models across different populations and settings. The number of studies related to OSA severity prediction is limited, indicating a need for more extensive research in this area. Encouraging and conducting additional studies on OSA severity prediction will advance the understanding of the factors influencing OSA progression and severity. This could include longitudinal studies and investigations in varied settings. The study acknowledges that OSA severity is a relatively new target, and more research is needed in larger and diverse settings. Continuing to explore the integration of machine learning techniques in digital healthcare for OSA and other sleep disorders and collaborating with healthcare professionals will ensure the applicability and effectiveness of these models in clinical practice.

## 5 Conclusion

In conclusion, the study evaluated four classification and grading prediction models for OSA, finding that LightGBM demonstrated better performance and displayed some grading ability for OSA severity. However, there is a recognized need for additional indicators to enhance the accuracy of these models. In the context of dichotomous screening prediction models, all five algorithms exhibited effective predictions for patients with suspected OSA. Notably, RF stood out with the best prediction effect, achieving an AUC of 0.98. The superior performance of RF suggests its potential for widespread adoption and promotion in clinical and community settings. The study highlighted the pivotal role of three variables—BMI, age, and drowsiness—in influencing the prediction results of OSA. Recognizing the significance of these variables underscores their crucial contribution to the accuracy and effectiveness of OSA prediction models.

## Data availability statement

The raw data supporting the conclusions of this article will be made available by the authors, without undue reservation.

## Ethics statement

The studies involving humans were approved by the Medical Ethics Committee of the Affiliated Hospital of Xuzhou Medical University, no. XYFY-KL341-01. The studies were conducted in accordance with the local legislation and institutional requirements. The participants provided their written informed consent to participate in this study.

## Author contributions

KL: Writing – original draft, Writing – review & editing, Data curation, Formal analysis, Investigation, Validation. SG: Formal analysis, Software, Writing – original draft. PZ: Data curation, Investigation, Methodology, Supervision, Writing – review & editing. WL: Investigation, Methodology, Supervision, Writing – review & editing. PS: Conceptualization, Data curation, Writing – original draft, Writing – review & editing. LZ: Conceptualization, Data curation, Writing – original draft, Writing – review & editing.
