# Supplementary material for: Development and application of a machine learning-based predictive model for obstructive sleep apnea screening
Source: Front Big Data. 2024 May 16;7:1353469. doi: 10.3389/fdata.2024.1353469 (PMC11137315; doi:10.3389/fdata.2024.1353469)
Supplement: Supplementary file 1 [file Data_Sheet_1.docx]

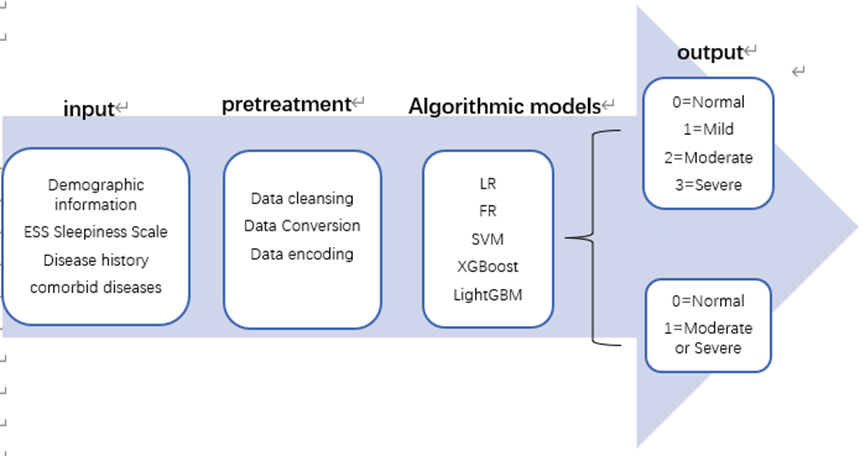


Figure 1: Prediction model flow chart

Table 1 Comparison of various parameters between groups with different degrees of OSA (four categories)

| **Parameters** | **Normal**（n=102） | **Mild**  （n=100） | **Moderate**（n=95） | **Severe**  (n=142) | **P** |
| --- | --- | --- | --- | --- | --- |
| Male | 53 | 77 | 71 | 126 | ＜0.001 |
| Age | 37（30,49） | 41（32，52） | 42（33,53） | 46（35,54） | 0.01 |
| BMI | 23.86±2.75 | 25.41±2.92 | 25.63±3.36 | 28.69±3.07 | ＜0.001 |
| Snoring | 81 | 96 | 92 | 139 | ＜0.001 |
| Waking up at night with suffocation | 30 | 50 | 50 | 83 | ＜0.001 |
| Morning headache | 14 | 21 | 20 | 32 | 0.359 |
| Loss of memory and concentration | 32 | 64 | 57 | 90 | ＜0.001 |
| Gastroesophageal reflux | 8 | 33 | 17 | 51 | ＜0.001 |
| Dry mouth | 46 | 71 | 74 | 111 | ＜0.001 |
| ESS total score | 0 | 17 | 23 | 61 | ＜0.001 |
| Hypertension | 9 | 22 | 16 | 52 | ＜0.001 |
| Coronary  heart disease | 4 | 3 | 2 | 10 | 0.248 |
| Arrhythmia | 7 | 8 | 3 | 10 | 0.525 |
| Thyroid disease | 6 | 6 | 8 | 3 | 0.177 |
| Cerebrovascular disease | 3 | 5 | 11 | 13 | 0.074 |

Table 2 Parameters of each model of the four classifications

| **Model** | **Parameters** |
| --- | --- |
| LR | penalty=l2,solver=lbfgs,C=1182 ,random_state=44 |
| SVM | kernel=linear,random_state=1,probability=True,decision_function_shape= ovr |
| RF | n_estimators=277,max_depth=6,min_samples_split=2,min_samples_leaf=10, random_state=98 |
| XGBoost | verbosity=0,alpha=0.65,subsample=0.22,colsample_bytree=0.25,  random_state=71, max_depth=6 |
| LightGBM | learning_rate=0.09 ,lambda_l2=0.98,num_leaves=2,feature_fraction=0.41,  min_child_samples=10, num_class=4, max_depth=-1 |

Table 3 Comparison of prediction performance of five models with four classifications

| Evaluation indicators | LightGBM | XGBoost | SVM | LR | RF |
| --- | --- | --- | --- | --- | --- |
| AUC | 0.76 | 0.69 | 0.75 | 0.76 | 0.73 |
| Accuracy | 0.78 | 0.70 | 0.75 | 0.76 | 0.77 |
| Sensitivity | 0.51 | 0.41 | 0.50 | 0.48 | 0.49 |
| Specificity | 0.85 | 0.81 | 0.84 | 0.84 | 0.84 |


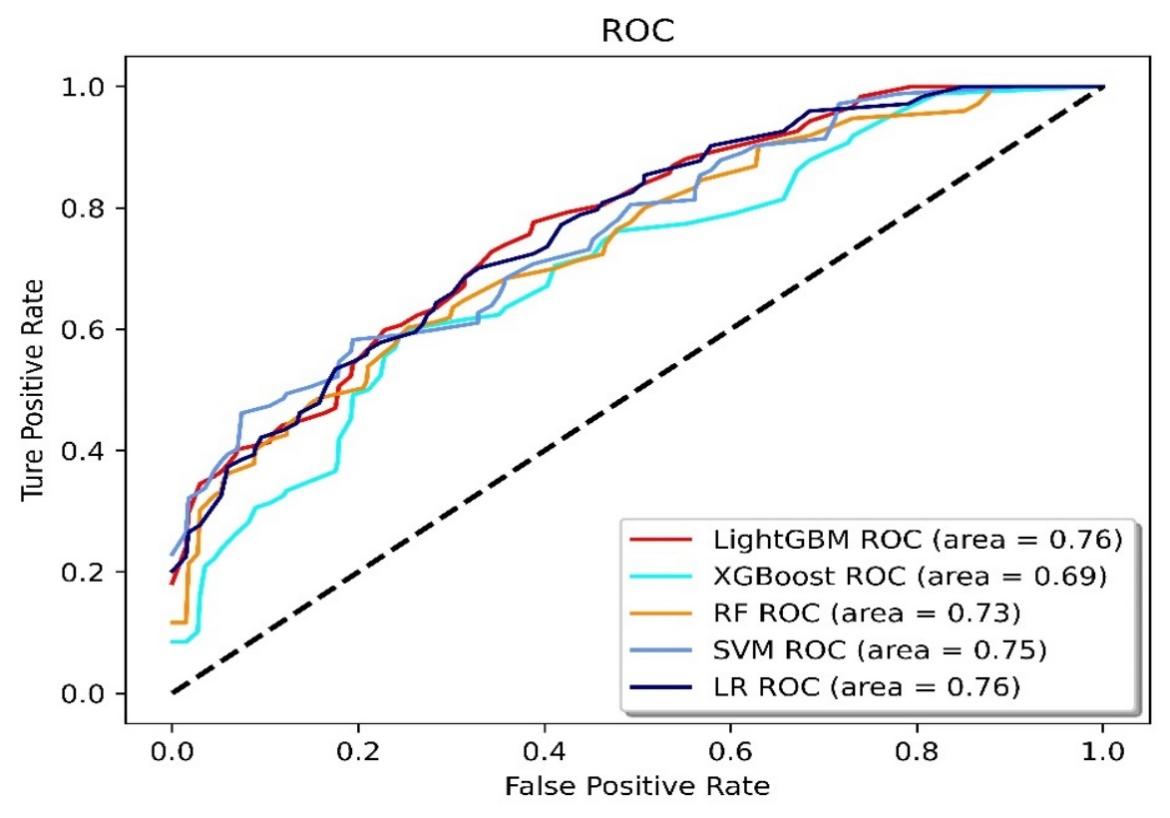


Fig.2 ROC curves of four classification and five models


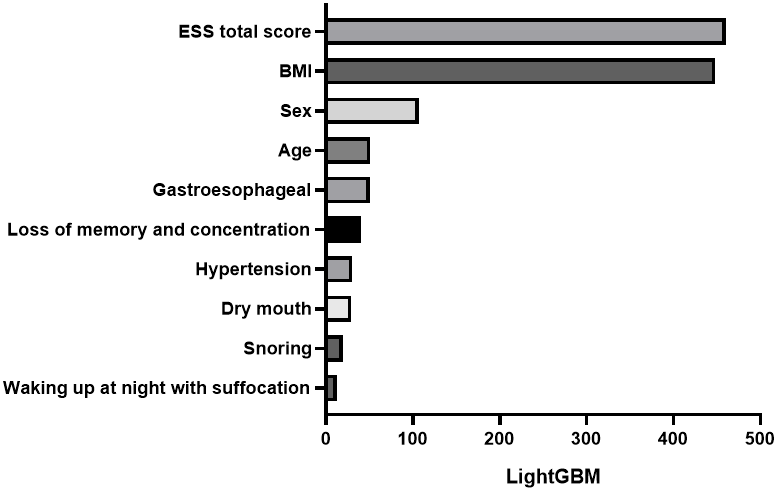

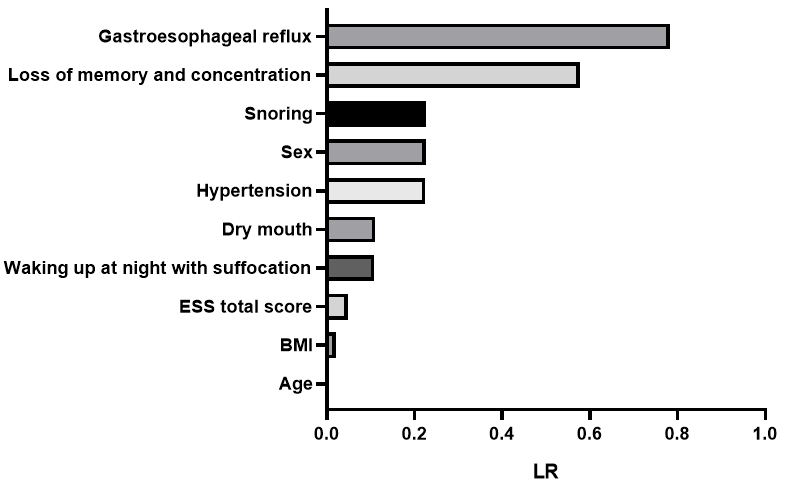


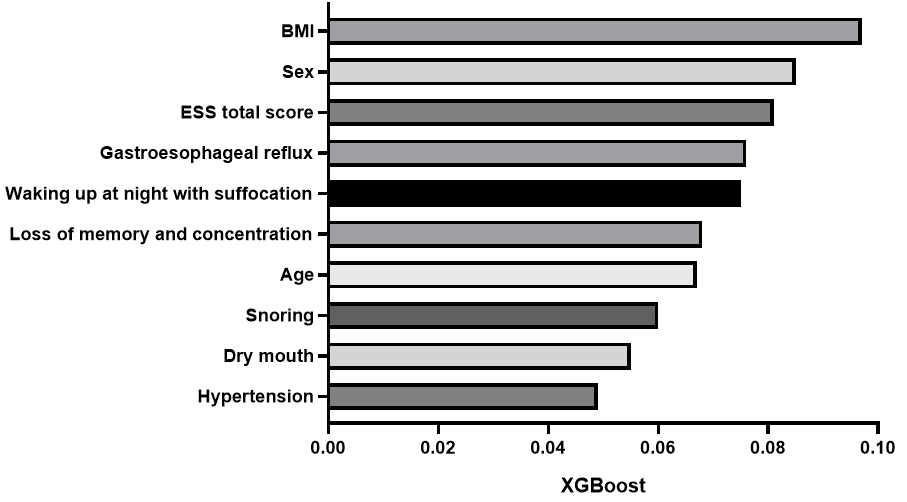

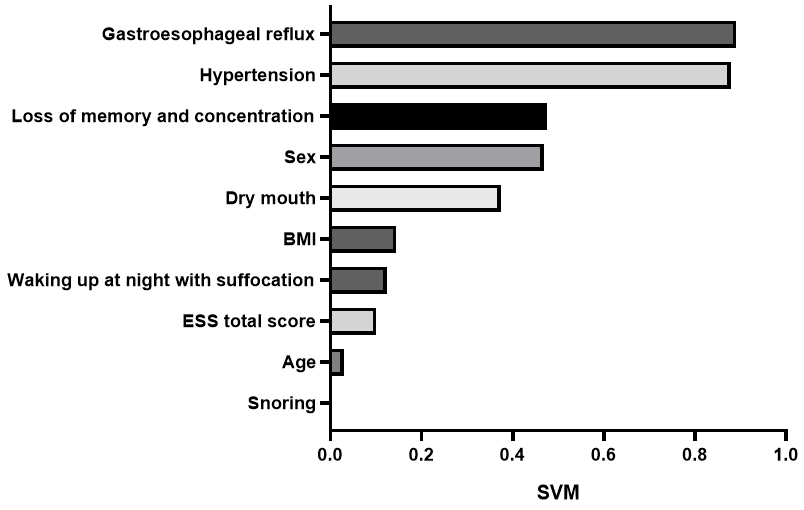

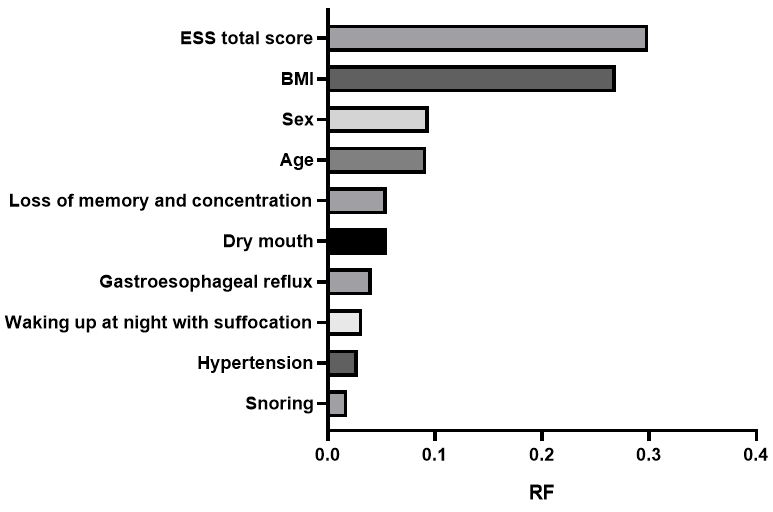


Fig.3 Ranking chart of the importance of variables of five algorithms in the four-classification OSA classification model

Table 4 Comparison of parameters between the non-OSA group and the moderate-to-severe OSA group (dichotomous classification)

| **parameters** | **non-OSA**  （n=102） | **OSA**  （n=237） | **P** |
| --- | --- | --- | --- |
| Male | 53 | 197 | ＜0.001 |
| Age | 37（30,49） | 44（34,54） | 0.001 |
| BMI | 23.86±2.75 | 27.47±3.52 | ＜0.001 |
| Snoring | 81 | 231 | ＜0,001 |
| Waking up at night with suffocation | 30 | 133 | ＜0.001 |
| Morning headache | 14 | 53 | 0.067 |
| Loss of memory and concentration | 32 | 147 | ＜0.001 |
| Gastroesophageal reflux | 8 | 68 | ＜0.001 |
| Dry mouth | 46 | 185 | ＜0.001 |
| ESS Total Score | 0 | 84 | ＜0.001 |
| Hypertension | 9 | 68 | ＜0.001 |
| Coronary heart disease | 4 | 12 | 0.649 |
| Arrhythmia | 7 | 12 | 0.509 |
| Thyroid disease | 6 | 11 | 0.631 |
| Cerebrovascular disease | 3 | 24 | 0.025 |

Table 5 Parameters of each model of binary classification

| **Model** | **Parameters** |
| --- | --- |
| LR | penalty=l2,solver=newton-cg,multi_class=multinomial |
| SVM | kernel=rbf,gamma=auto,degree=1,cache_size=5000,probability=True |
| RF | random_state=2022,max_depth=3,n_estimators=10 |
| XGBoost | random_state=2022,verbosity=0,max_depth=3,learning_rate=0.01, n_estimators=100 |
| LightGBM | random_state=2022,max_depth=3,learning_rate=0.1,n_estimators=200 |

Table 6 Comparison of prediction performance of five binary classification models

| Evaluation indicators | LightGBM | XGBoost | SVM | LR | RF |
| --- | --- | --- | --- | --- | --- |
| AUC | 0.95 | 0.94 | 0.91 | 0.95 | 0.97 |
| Accuracy | 0.87 | 0.85 | 0.88 | 0.88 | 0.91 |
| Sensitivity | 0.92 | 0.84 | 0.94 | 0.90 | 0.94 |
| Specificity | 0.71 | 0.82 | 0.70 | 0.82 | 0.82 |


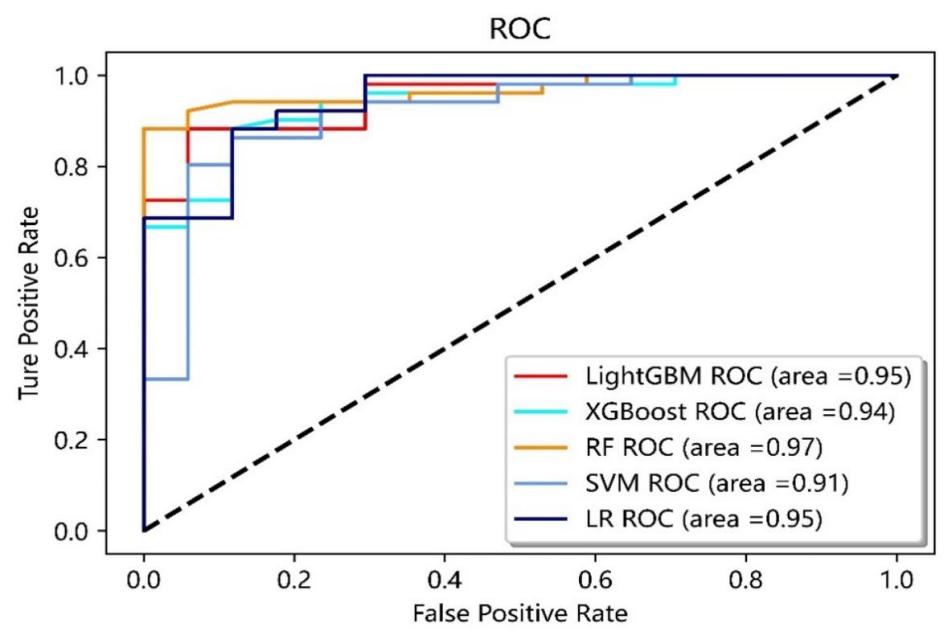


Figure 4: ROC curves of the five models of binary classification


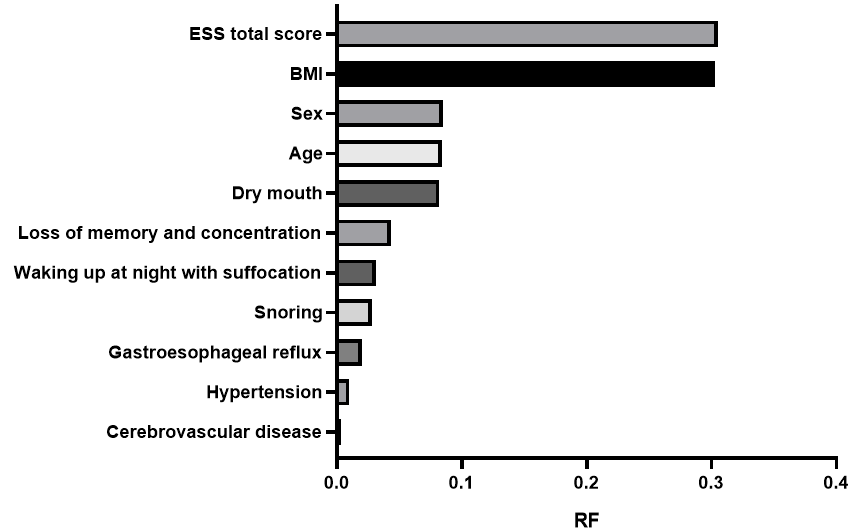

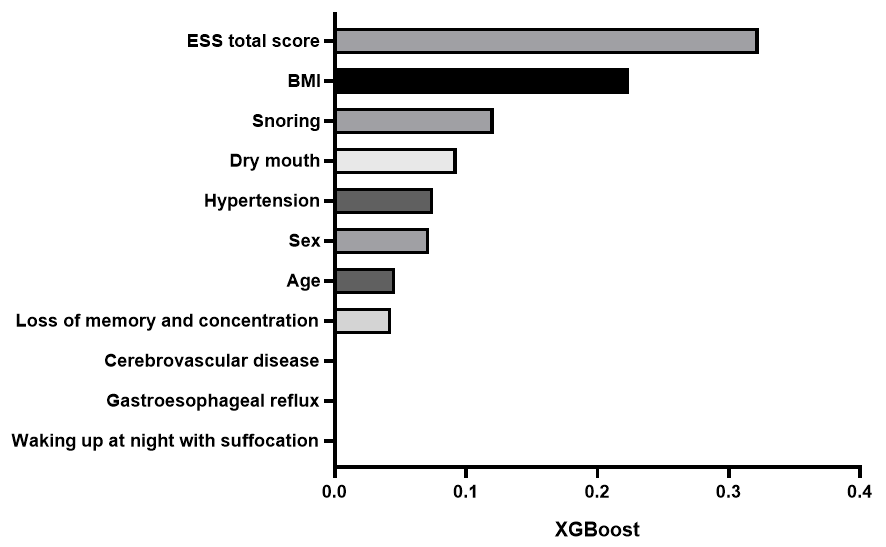

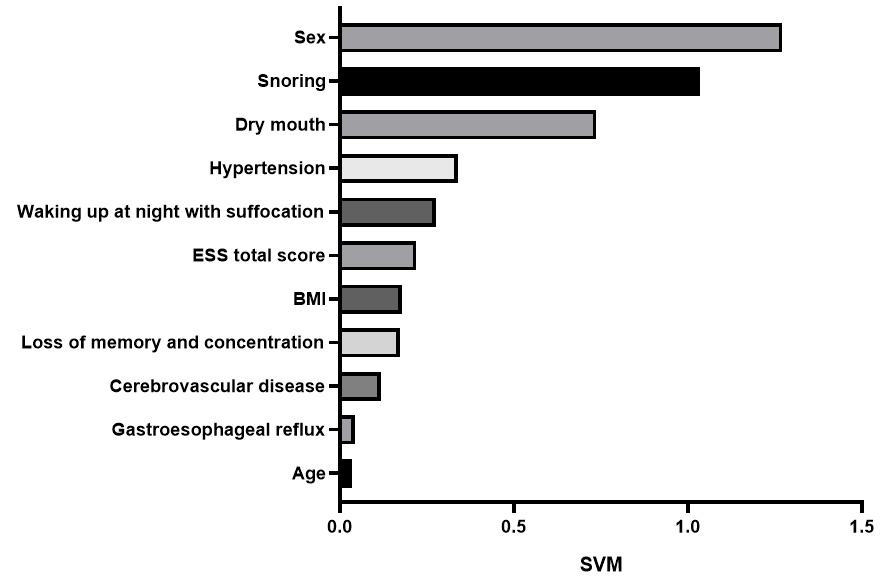

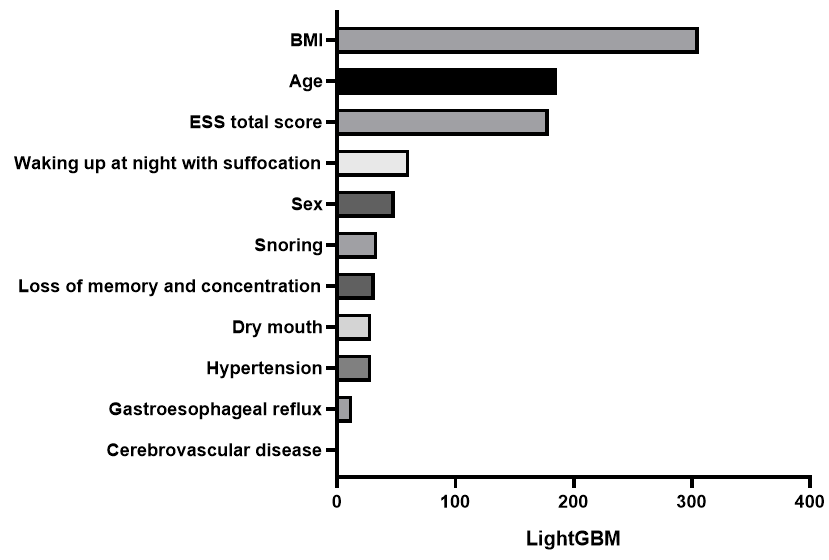


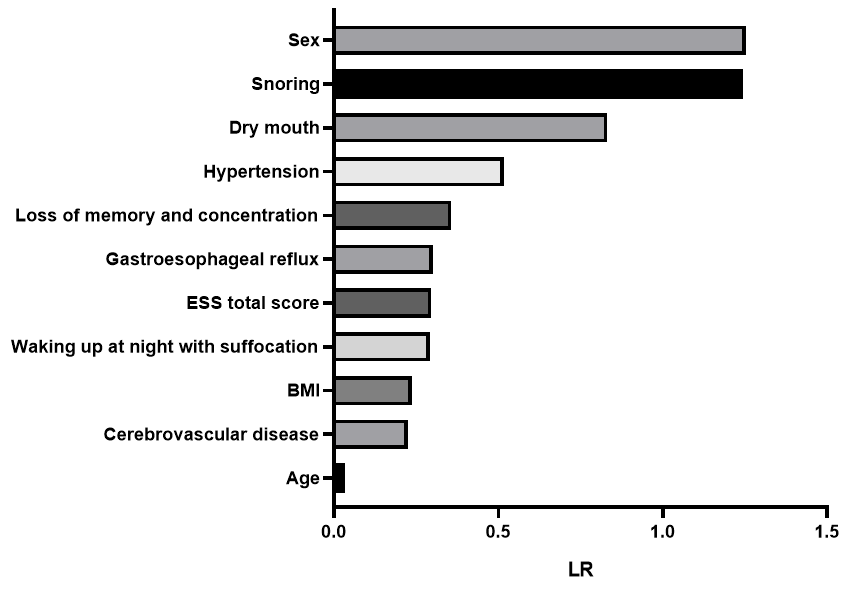


Fig.5 Ranking chart of the importance of variables of five algorithms of the binary classification OSA screening model
